# Treatment-related pure large-cell neuroendocrine carcinoma of the prostate with systemic metastases in a young adult: a rare case report

**DOI:** 10.3389/fonc.2025.1617699

**Published:** 2025-07-07

**Authors:** Baolin Zhang, Yifan Zhu, Renpeng Huang, Feng Zhou, Zhixin Ling

**Affiliations:** ^1^ Department of Urology, The First Affiliated Hospital of Soochow University, Suzhou, Jiangsu, China; ^2^ Department of Pathology, First Affiliated Hospital of Soochow University, Suzhou, Jiangsu, China

**Keywords:** prostate cancer, large cell, neuroendocrine carcinoma, metastasis, case report

## Abstract

Large cell neuroendocrine carcinoma of the prostate is an extremely rare malignant subgroup with limited reported cases. Little is known about its pathological characteristics, treatment options and long-term prognosis. In this case, we report a young patient presenting with painless gross hematuria for 3 months, accompanied with elevated serum total prostate-specific antigen (tPSA) level of 83.7 ng/ml. Magnetic resonance imaging (MRI) and 18F-FDG Positron Emission Tomography - Computed Tomography (¹^8^F-FDG PET/CT) indicated giant prostate mass, which metastasized to bilateral lungs, bones and lymph nodes. Prostate biopsy and transurethral resection of the prostate confirmed the diagnosis of adenocarcinoma with a Gleason score of 4 + 5. After receiving 12 months of goserelin acetate, rezvilutamide and six cycles of docetaxel, the patient further underwent laparoscopic radical prostatectomy (LRP). Immunohistochemical analysis (Syn+/CgA+/AR−) combined with treatment history revealed a histopathological diagnosis of treatment-related LCNEPC. Subsequently, the patient then received immunotherapy with serplulimab (300 mg) and the EP regimen (combining etoposide and cisplatin) chemotherapy. After six cycles of chemo-immunotherapy, further examination indicated reduction in size of multiple lymph nodes and lung metastases by March 2025. Here we reported a rare case of treatment-related LCNEPC, who had experienced systematic therapy with comprehensive care. These diagnostic and therapeutic approaches may improve the management capability and highlight the critical role of multimodal strategies in the subsequent cases.

## Introduction

Prostate cancer is the most common malignant neoplasm among men in the United States, with the second-highest mortality rate. Neuroendocrine prostate cancer (NEPC) is a rare and aggressive subtype of prostate cancer. It accounts for around 0.5% to 2% of all prostate cancer cases at initial diagnosis (*de novo* NEPC) (1). The 2022 World Health Organization (WHO) classification categorizes neuroendocrine neoplasms of the prostate into well-differentiated neuroendocrine tumors (NETs) and poorly differentiated neuroendocrine carcinomas (NECs) (2). Poorly differentiated NECs are divided into two aggressive subtypes: small cell neuroendocrine prostate carcinoma (SCNEPC) and large cell neuroendocrine prostate carcinoma (LCNEPC). Although platinum-based regimens, such as cisplatin or carboplatin combined with etoposide, have proven effective in the majority of patients with LCNEPC. However, the durability of these responses remains limited, with rapid disease progression often observed following initial tumor regression (3). In this case, a 55-year-old man presented with painless gross hematuria for 3 months. Imaging examinations and surgical pathological results further confirmed a diagnosis of prostate adenocarcinoma with multiple metastases to the lungs and bones. Subsequently, goserelin acetate plus rezvilutamide endocrine therapy combined with 6 cycles of docetaxel chemotherapy was conducted. Then, in September 2024, the patient underwent LRP due to progressively worsening urinary difficulty; despite maintaining a PSA level below 0.05 ng/ml. Postoperative pathology confirmed a diagnosis of pure LCNEPC. The patient further received six rounds of EP regimen chemotherapy and serplulimab immunotherapy. Follow-up imaging in March 2025 demonstrated a partial reduction in size of lymph nodes and lung metastases; however, the overall disease progression indicated multi-system dissemination. Accordingly, applying evidence-based and guideline-directed therapies across different stages of prostate cancer has the potential to enhance clinical outcomes.

## Case description

**Figure 1:**
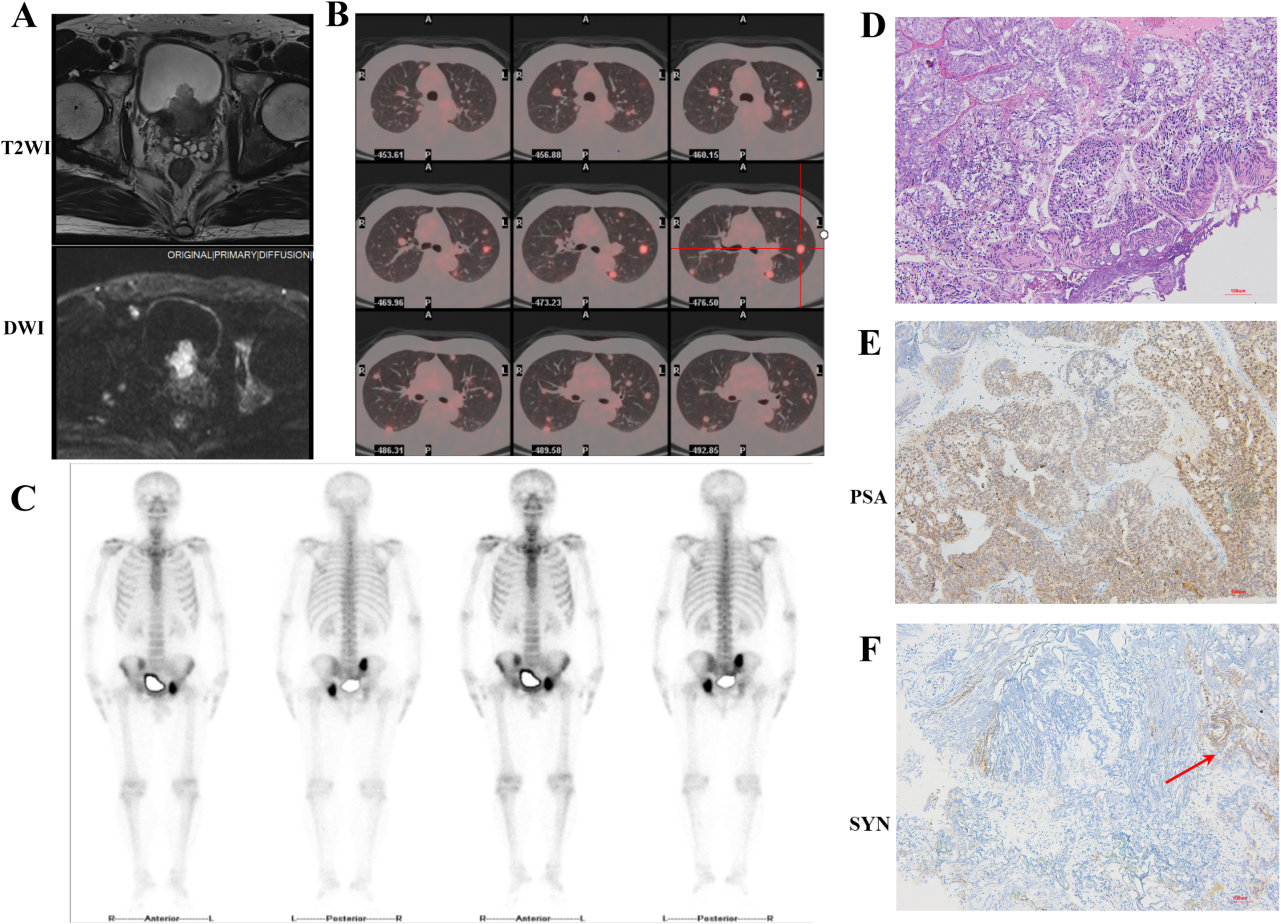
Imaging and pathological findings obtained during the patient’s initial hospitalization. **(A)** T2WI combined with DWI MRI images revealed prostate tumor invasion to the bladder. **(B)** ¹^8^F-FDG PET-CT images showed multiple metastases in both lungs. **(C)** SPECT whole-body bone scan indicated multiple metastases in the right ilium, left acetabulum, and ischium. **(D)** HE staining. The glandular architecture is disrupted. Tumor cells exhibited enlarged, hyperchromatic nuclei with prominent nucleoli and increased nuclear-cytoplasmic ratio (10×). **(E, F)** Immunohistochemical staining revealed strong positive expression of PSA and scattered SYN expression positivity (10×).

The 55 years-old man was admitted to the department of urology on July 6, 2023 due to painless gross hematuria for 3 months. Prior to admission, a non-contrast whole-abdominal computed tomography (CT) scan demonstrated enlargement of the prostate with a soft tissue density shadow along the posterior bladder wall, and findings suggestive of prostatic carcinoma with possible invasion into the bladder. The patient had no symptoms such as dysuria, bone pain or dyspnea, and no history of renal or pulmonary diseases. The serum tPSA level was 83.737 ng/mL; however, digital rectal examination (DRE) has not detected any palpable solid nodules within the prostate. Following admission, contrast-enhanced prostate MRI revealed extensive lesions involving both the peripheral and transitional zones, with evidence of extracapsular extension beyond the adjacent prostatic capsule. T2-weighted images further confirmed tumor infiltration into bilateral posterior wall of bladder, alongside multiple metastasis lesions in the bilateral iliac bone and iliac acetabulum. PI-RADS score was 5 (**Figure 1A**). Moreover, we conducted an ¹^8^F-FDG PET/CT, a single-photon emission computed tomography (SPECT) whole-body bone scan, and a contrast-enhanced chest CT. These radiological findings provided further confirmation of prostatic malignancy with local invasion into the bladder, along with metastatic spread to both lungs, multiple pelvic bones, and pelvic lymph nodes (**Figures 1B, C**). According to the eighth edition of the American Joint Committee on Cancer (AJCC) staging system, the clinical stage is classified as stage IV (T4N1M1c). Based on the LATITUDE criteria for high-risk disease and the CHAARTED criteria for high tumor burden, T4N1M1c is categorized as high-risk and high tumor burden metastatic hormone-sensitive prostate cancer (mHSPC).

Due to intractable hematuria symptoms caused by the prostate tumor, the patient underwent transurethral resection of the prostate (TURP) and bladder tumor on July 13, 2023. Concurrently, due to the large-volume and extensive nature of the prostate tumor, a systematic prostate biopsy combined with MRI/US fusion-targeted biopsy was performed. Histopathological examination of both the transurethral resection specimen and the transperineal prostate needle biopsy tissue revealed prostate adenocarcinoma, with a Gleason score of 4 + 5 = 9, WHO/ISUP group 5 (**Figures 1D, E**). It should be emphasized that the pathologist examined all transurethral resection specimens and biopsy samples, which consistently showed morphologically uniform acinar adenocarcinoma, with no evidence of neuroendocrine tumor components. CHART trial demonstrated that rezvilutamide plus androgen deprivation therapy (ADT) significantly improved radiographic progression-free survival (rPFS) and overall survival (OS) compared to bicalutamide plus ADT in patients with high-volume mHSPC (4). Then, the patients immediately received ADT with second generation anti-androgen therapy after operation (goserelin acetate 3.6mg per month and rezvilutamide 240mg daily). From August 4, 2023 to November 21, 2023, six cycles of docetaxel plus prednisone chemotherapy were performed owing to extensive tumor burden with multiple metastatic sites.

**Figure 2:**
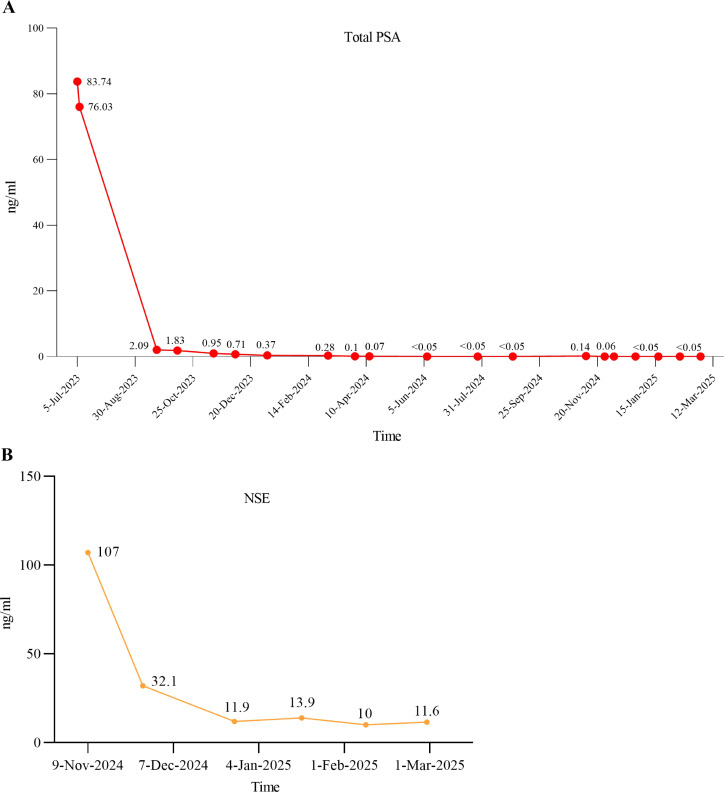
The serum levels of tPSA and NSE after therapy. **(A)** The tPSA level changes after receiving standard ADT combined with rezvilutamide and docetaxel chemotherapy; **(B)** The NSE level changes after cisplatin and etoposide chemotherapy in combination with serplulimab immunotherapy.

On April 12, 2024, the patient was readmitted to the hospital for examination. MRI demonstrated a reduction in prostate tumor volume and pelvic bone lesions following systemic therapy, as compared to MRI findings obtained on July 10, 2023. The prostate-specific membrane antigen positron emission tomography/computed tomography (PSMA PET/CT) also indicated post-treatment changes in the primary prostate lesion, accompanied by multiple metastatic foci in the bilateral lungs, the left 6th rib, the 5th lumbar vertebra, the bilateral hip bones, and the right iliac bone. Although the imaging results did not show a significant reduction in metastatic lesions, no significant progression was observed either. The patient also received regular teste for tPSA during the endocrine therapy which was controlled less than 0.05ng/ml (**Figure 2A**). Despite the patient’s PSA remaining at a very low level, the patient developed worsening dysuria and urinary difficulty.

**Figure 3:**
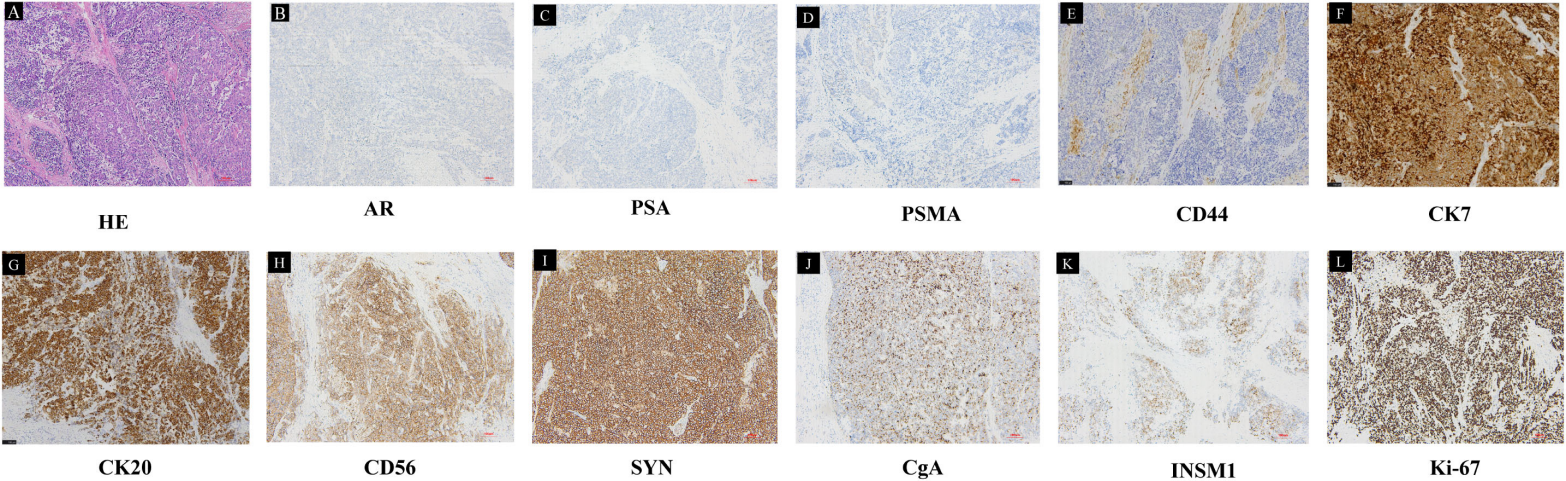
Pathological results of the LRP specimen. **(A)** HE staining showed the tumor cells were arranged in large, solid nests, exhibiting necrosis and a characteristic palisading pattern of nuclei at the periphery of the nests. Tumor cells had abundant cytoplasm, a relatively lower nuclear-to-cytoplasmic ratio, and prominent nucleoli. IHC was negative for **(B)** AR, **(C)** PSA, **(D)** PSMA, **(E)** CD44, and positive for **(F)** CK7, **(G)** CK20, **(H)** CD56, **(I)** SYN, **(J)** CgA, **(K)** INSM1, **(L)** Ki-67 (10×).

On August 29, 2024, the patient was readmitted to the hospital again due to recurrent urinary tract infection accompanied by dysuria and hematuria persisting for two months. More importantly, the patient’s hemoglobin level decreased to 76 g/L, and the patient developed acute urinary retention one week prior to hospital admission. DRE revealed that both lobes of the prostate were palpable as hard, enlarged masses. B-ultrasound indicated that within a short period of four months, the residual prostate tumor had rapidly increased in size and was protruding into the bladder, with the prostate measuring approximately 50 × 50 × 59 mm. Given the patient’s PSA remaining at a low level, the rapidly enlarging prostate tumor, and recurrent symptoms of urinary difficulty, the possibility of a neuroendocrine tumor is strongly considered. To address the anemia caused by severe hematuria, surgical intervention was prioritized as the primary treatment option. Conservative measures, such as suprapubic cystostomy, could not address the underlying cause of persistent hematuria. Additionally, the patient expressed concerns about the quality of life associated with long-term catheter use and the potential risk of secondary infections, thus favoring surgical management. Based on our prior experience, we have managed multiple cases of hematuria induced by castration-resistant prostate cancer using transurethral resection of the prostate (TURP) to control the disease. However, patients often experienced recurrent hematuria within a short period. Therefore, considering our clinical experience, the goals of controlling hematuria and tumor burden, and the patient’s quality-of-life preferences, laparoscopic radical prostatectomy (LRP) was ultimately selected. Then the patient underwent LRP on September 3, 2024. Intraoperatively, the prostate was found to be significantly enlarged, with the tumor exhibiting clear invasion into the bladder. The tumor margin was in close proximity to the bilateral ureteral orifices, particularly the right ureter. Postoperative routine pathological examination revealed pure treatment-related LCNEPC, with no adenocarcinoma component identified in the specimens. Hematoxylin-eosin (HE) staining showed cancer cells with abundant cytoplasm, accompanied by comedo-like necrosis (**Figure 3A**). Immunohistochemistry (IHC) demonstrated positive expression of cytokeratin (CK), CK20, and CK7, while negative expression was observed for PSA, androgen receptor (AR), and CD44. Scattered positive expression was noted for chromogranin A (CgA) and insulinoma-associated protein 1 (INSM1), with diffuse positive expression for synaptophysin (SYN) and CD56. Moreover, Ki-67 exhibited strong positive expression (95%) (**Figures 3B-L**). Concurrently, genetic sequencing was also performed on the tumor tissue, which indicated somatic mutations in HRAS and P53, as well as a pathogenic germline FANCA p.F831Sfs*4 variant associated with homologous recombination repair (HRR). Poly (ADP-ribose) Polymerase (PARP) inhibitors have demonstrated promising results in FANCA-altered metastatic castration-resistant prostate cancer (mCRPC) (5–7), suggesting their potential efficacy in this context.

Two months following surgery, the patient presented with acute renal insufficiency, evidenced by an elevation in serum creatinine levels to 784.9 µmol/L, in contrast to a postoperative follow-up value of 63 µmol/L. Ultrasonography revealed bilateral hydronephrosis. Subsequently, the patient underwent bilateral percutaneous nephrostomy procedures, resulting in a rapid restoration of normal renal function. Starting on November 15, 2024, the patient started to receive serplulimab (300 mg) for immunotherapy, together with chemotherapy consisting of etoposide (0.1 mg, administered on days 1–4) and cisplatin (35 mg, administered on days 1–3). Following six cycles of combined immuno-chemotherapy, the neuron-specific enolase (NSE) level decreased significantly from an initial value of 107 ng/ml to 11.6 ng/ml (**Figure 2B**). On December 27, 2024, the patient conducted a whole-body contrast-enhanced CT scan to assess for metastatic disease. Imaging revealed multiple lymph node metastases in the bilateral pelvic region, left iliac vessels, and retroperitoneum, as well as suspected metastatic lesions in the spine, pelvis, and bilateral ribs. Additional findings included left adrenal gland metastasis, and bilateral pulmonary metastases. On March 26, 2025, the patient underwent the same examination again to evaluate disease status following six cycles of combined immunotherapy and chemotherapy. Imaging demonstrated a reduction in the size of lymph node metastases in the bilateral pelvic region, adjacent to the left iliac vessels, and in the retroperitoneum compared to prior imaging. Pulmonary metastases also showed a marked decrease in size; however, the number of skeletal metastases had increased. Overall, disease progression was controlled relative to earlier assessments; however, the condition persisted as widespread multi-system dissemination (**Table 1**).

**Table 1:** Timeline of management.

Time line in management	
July 6^th^ ,2023	-First admission hospital
July 14^th^ ,2023	-Transurethral resection of the prostate and bladder tumor, concurrently with a transperineal prostate needle biopsy
August, 2023- September, 2024	-Androgen deprivation therapy and rezvilutamide
August, 2023- November ,2023	-Completed 6 cycles of docetaxel plus prednisone chemotherapy
September 4^th^, 2024	-Laparoscopic radical prostatectomy
November 9^th^, 2024	-Bilateral percutaneous nephrostomy
November, 2024- March, 2025	-Received six rounds of cisplatin and etoposide chemotherapy in combination with serplulimab immunotherapy
March,2025-now	- Subsequently scheduled for immunotherapy and radiology ± PARP inhibitor

## Discussion

Neuroendocrine cells are dispersed throughout the prostatic glands across all anatomic zones, constituting less than 1% of the benign prostatic glandular epithelium (8). In normal human prostate, neuroendocrine cells are more common in transition zone and peripheral zone than in central zone, indicating its potential role in the development of benign prostatic hyperplasia (BPH) and prostate cancer (9, 10). However, their functions and roles are still largely unknown, though they may be involved in regulating the growth, differentiation and secretory function of the prostate gland (11).

Androgen deprivation therapy (ADT) is considered as a standard and effective treatment for primary metastatic castration-sensitive prostate cancer; however, the disease will inevitably progress to castration-resistant prostate cancer (CRPC) stage within approximately two years (12, 13). Neuroendocrine prostate cancer (NEPC) is a rare and aggressive subtype of CRPC, with its prevalence increases significantly following treatments with second-generation androgen pathway inhibitors such as enzalutamide, abiraterone and apalutamide. Studies suggest that treatment-related neuroendocrine prostate cancer (t-NEPC) may develop in 15% to 25% of metastatic CRPC cases following ADT or other hormonal treatments (14, 15).

Though, the origin of NEPC remains incompletely understood, there are several main mechanisms of NEPC development that have been reported in previous studies. Some researchers believe that the normal NE cells situated in the normal prostate gland could be selected for survival during ADT and ultimately expand (11, 16, 17). Other studies illustrate that transdifferentiation from adenocarcinoma into NEPC after endocrine therapy is more convinced mechanism. This process, often termed as t-NEPC, always involves linage plasticity which refers to a shift cellular phenotype from androgen receptor-dependent adenocarcinoma to androgen receptor-independent neuroendocrine carcinoma (18, 19). Some recent studies also hypothesize that NEPC may arise from luminal cells or from basal cells that lose their basal features and acquire luminal-like characteristics (20–22). As for our case, we found that the patient progressed to pure LCNEPC after endocrine therapy within only 12 months. The patient’s PSA level decreased rapidly from 76.03 ng/mL to 2.1 ng/mL after two months of ADT and antiandrogen therapy and subsequently remained at a relatively low level (**Figure 2A**). However, imaging in April 2024 revealed that the volume of the residual prostatic tumor and distant metastatic lesions had not significantly decreased compared to the postoperative period. This raises the possibility that the prostate cancer may have undergone gradual transdifferentiation into a neuroendocrine phenotype during this period. On the other hand, immunohistochemical analysis of the initial TURP specimen revealed relatively high expression of synaptophysin in a subset of tumor cells (**Figure 1F**), indicating neuroendocrine differentiation within part of the tumor. Therefore, the possibility that these NE cells were selected under treatment pressure and subsequently expanded to develop into NEPC cannot be excluded. Alternatively, it is also possible that both mechanisms occurred simultaneously.

As LCNEPC is extremely rare, its pathological diagnosis remains challenging. Histologically, the tumor cells are arranged in large, solid nests, often exhibiting necrosis and a characteristic palisading pattern of nuclei at the periphery of the nests (23). In contrast to SCNEPC, LCNEPC is distinguished by more abundant cytoplasm, a relatively lower nuclear-to-cytoplasmic ratio, vacuolated nuclear chromatin, and prominent nucleoli. Immunohistochemically, LCNEPC consistently expresses one or more neuroendocrine markers, such as SYN, chromogranin A (CgA), or CD56 (24). Additionally, it demonstrates variable expression of cytokeratins, including cytokeratin 7 (CK7) or cytokeratin 20 (CK20), reflecting its heterogeneous differentiation profile. However, the expression of PSA and PAP are always negative (25). These features indicate morphological and molecular characteristics of LCNEPC (26). As is shown in the case description, the tumor was composed of pure neuroendocrine cells without any component of adenocarcinoma. The tumor cells were arranged in large nests, exhibiting abundant cytoplasm and prominent comedo-like necrosis. Moreover, IHC demonstrated diffuse positive expression of SYN, CD56, and Ki-67 (95%), while PSA, AR, and PSMA were all negative (**Figures 3B-L**). Based on the integration of clinical history and pathological findings, a diagnosis of treatment-related LCNEPC was confirmed by the pathologists. In addition, we consulted Professor Jiaoti Huang, the chairman of the department of pathology in Duke University, whose professional expertise further confirmed the pathological findings.

Until now, there are no standard guidelines concerning the therapeutic approaches managing NEPC. Platinum-based chemotherapy is a common therapy option for patients with pure small cell carcinoma based on small cell lung carcinoma data. A Phase II clinical trial evaluated the efficacy and safety of a combination regimen comprising doxorubicin, etoposide, and cisplatin in patients with SCNEPC. Though 22 patients (61%) showed partial response; toxicity was severe and three patients died of side effects. Therefore, addition of doxorubicin to the etoposide/cisplatin regimen in not recommended for patients with SCNEPC (27). Zhu et al. investigated the treatment strategies and survival outcomes of 510 patients with NEPC, and he noted that chemotherapy was the most effective therapy, which increased the OS of patients with regional (distant) metastases from 8 months (5 months) to 13.5 months (9 months) (28). As for LCNEPC, including De Nove and treatment-related LCNEPC, a total of 14 patients among 24 reported cases received cisplatin/carboplatin-based chemotherapy as part of their treatment regimen (29, 30). In addition to chemotherapy, immune checkpoint inhibitors may also be an important therapeutic supplement based on small cell lung carcinoma data (31, 32). Another unpublished case which was reported in the Chinese Urological Association meeting also indicated that toripalimab combined with cisplatin-based chemotherapy had satisfied efficacy on a patient with SCNEPC. In this case, the patient initially refused further chemotherapy until he developed bilateral hydronephrosis due to tumor invasion of the ureter. Subsequently, he began to receive serplulimab for immunotherapy, together with chemotherapy consisting of etoposide and cisplatin. At a minimum, the tumor burden has decreased, and the disease has been partially controlled. Therefore, immunotherapy combined with platinum-based chemotherapy may represent an effective treatment strategy for NEPC, although its potential to significantly prolong patient survival may be limited.

As a widely accepted consensus, radical prostatectomy is not considered suitable for patients with advanced metastatic prostate cancer. Therefore, the clinical value of surgical intervention in patients with metastatic CRPC remains controversial. A recent study systematically analyzed the patient demographics, survival rates, and treatment methods for SCNEPC and LCNEPC from the SEER database, which included a total of 718 cases of NEPC patients for further analysis. The 1-year cause-specific survival was 31.7% (95% C.I. 23.7–39.7) for combination therapy (surgery, radiation, and chemotherapy), 24.0% (95% C.I. 19.9–29.9) for surgery only with unknown chemotherapy status, 12.0% (95% C.I. 7.7–16.3) for surgery with chemotherapy, and 15.5% (95% C.I. 13.2–17.8) for chemotherapy alone (33). Therefore, radical prostatectomy also plays a vital role in prolonging the patients’ survival beyond chemotherapy. In this case, the patient experienced persistent hematuria and dysuria due to the rapid progression of NEPC. Chemotherapy could not quickly alleviate these symptoms, and the patient’s hemoglobin level was rapidly declining. Radical prostatectomy not only rapidly alleviated these symptoms, corrected anemia, but also provided a pathological diagnosis of NEPC that served as a basis for subsequent early intervention. Moreover, the patient is satisfied with the life quality after the surgery. Our experience suggests that surgery can serve as an individualized treatment option.

**Figure 4:**
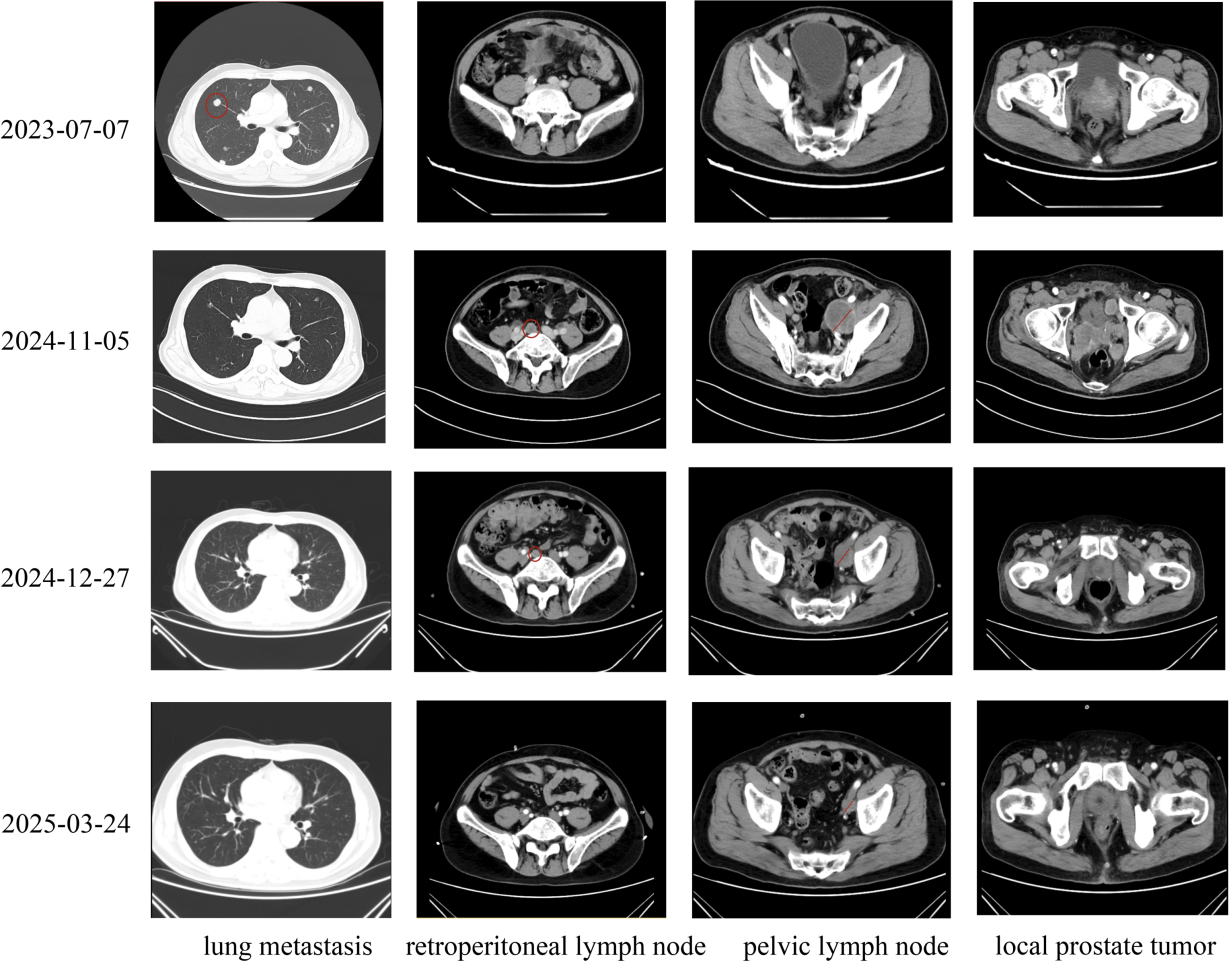
Timeline of Imaging Changes in Prostate Cancer Metastases Across Diagnosis, Treatment, and Follow-Up: On July 6, 2023, enhanced computed tomography (CT) scans of the chest, abdomen, and pelvis revealed multiple metastatic lesions in the lungs, locally advanced prostate tumors protruding into the bladder, small metastatic lymph nodes in the pelvis, and no evidence of retroperitoneal lymph node involvement in a patient diagnosed with prostate cancer. Following laparoscopic radical prostatectomy in September 2024, the patient declined immediate adjuvant chemotherapy. Subsequent enhanced CT scans performed on November 6, 2024, demonstrated rapid tumor progression, with enlarged pelvic lymph nodes, tumor recurrence in the surgical bed, and new metastatic lymph nodes in the retroperitoneum. Notably, pulmonary metastases had largely resolved following prior treatment. After initiating two cycles of cisplatin and etoposide chemotherapy combined with immunotherapy, enhanced CT scans on December 27, 2024, showed a marked reduction in the size of pelvic and retroperitoneal lymph nodes and tumor tissue in the surgical bed, indicating a significant therapeutic response. Following completion of six cycles of combined chemotherapy and immunotherapy, enhanced CT scans on March 24, 2025, revealed near-complete resolution of pulmonary metastases, retroperitoneal lymph nodes, and tumors in the surgical bed, with further reduction in left pelvic lymph nodes, confirming a robust and sustained treatment response.

Our case of treatment-related large cell neuroendocrine prostate carcinoma (LCNEPC) exhibits a distinct therapeutic approach compared to the 12 treatment-related LCNEPC cases reported by Nguyen et al. (29). First, our patient progressed from adenocarcinoma to LCNEPC in only 12 months, markedly faster than the over two-year progression observed in most cases reviewed by Nguyen et al., highlighting the aggressive nature of the disease. Second, while Nguyen et al.’s cases primarily utilized androgen deprivation therapy (ADT) alone, often combined with surgery or radiotherapy, our patient received guideline-directed triple therapy with ADT, rezvilutamide, and docetaxel for initial adenocarcinoma management. Third, following LCNEPC confirmation via laparoscopic radical prostatectomy (LRP) prompted by rapid tumor growth and urinary obstruction, our multidisciplinary team employed cisplatin/etoposide with serplulimab immunotherapy, diverging from the cisplatin/carboplatin-based chemotherapy commonly described by Nguyen et al. Fourth, follow-up CT imaging revealed near-complete resolution of pulmonary metastases and significant reduction in pelvic lymph node metastases, underscoring the efficacy of our sequential regimen. Additionally, genomic profiling identified a FANCA mutation, suggesting potential PARP inhibitor use, a strategy not reported by Nguyen et al. This tailored multimodal approach, integrating surgery, chemo-immunotherapy, and prospective targeted therapy, emphasizes the need for adaptive strategies to manage the rapid progression of treatment-related LCNEPC. Our multimodal treatment strategy demonstrated effectiveness against large cell neuroendocrine carcinoma, as evidenced by significant tumor regression observed in follow-up imaging (**Figure 4**).

As is known, LCNEPC is rare and highly aggressive subtype of prostate cancer, characterized by poor prognosis, rapid disease progression, and widespread metastatic dissemination. Due to the absence of reliable biomarkers for early detection and the lack of standardized, effective treatment strategies, LCNEPC remains a highly lethal malignancy. In this study, we report a case of a young patient diagnosed with prostate cancer accompanied by multiple metastases. The patient initially underwent TURP to reduce local tumor burden and alleviate symptoms of dysuria. Despite receiving standard ADT combined with rezvilutamide and docetaxel chemotherapy, the disease continued to progress. Subsequent radical prostatectomy confirmed a pathological diagnosis of pure LCNEPC. The patient was then treated with cisplatin and etoposide chemotherapy in combination with serplulimab immunotherapy. Remarkably, the patient has survived for 21 months since the initial diagnosis. Therefore, systematic treatment of prostate cancer at various stages, in accordance with clinical guidelines and evidence-based medicine, can improve patient outcomes. Given the identification of a somatic mutation in the FANCA gene through genomic profiling, local radiotherapy in combination with PARP inhibitors may represent a potential therapeutic approach moving forward.

## Data Availability

The original contributions presented in the study are included in the article/**Supplementary Material**. Further inquiries can be directed to the corresponding authors.
