# Supplementary material for: Treatment-related pure large-cell neuroendocrine carcinoma of the prostate with systemic metastases in a young adult: a rare case report
Source: Front Oncol. 2025 Jul 7;15:1617699. doi: 10.3389/fonc.2025.1617699 (PMC12277134; doi:10.3389/fonc.2025.1617699)
Supplement: Supplementary file 1 [file Table1.docx]

| Time line in management | |
| --- | --- |
| July 6^th^ ,2023 | -First admission hospital |
| July 14^th^ ,2023 | -Transurethral resection of the prostate and bladder tumor, concurrently with a transperineal prostate needle biopsy |
| August, 2023- September, 2024 | -Androgen deprivation therapy and rezvilutamide |
| August, 2023- November ,2023 | -Completed 6 cycles of docetaxel plus prednisone chemotherapy |
| September 4^th^, 2024 | -Laparoscopic radical prostatectomy |
| November 9^th^, 2024 | -Bilateral percutaneous nephrostomy |
| November, 2024-  March, 2025 | -Received six rounds of cisplatin and etoposide chemotherapy in combination with serplulimab immunotherapy |
| March,2025-now | - Subsequently scheduled for immunotherapy and radiology ± PARP inhibitor |
